# The prevalence, awareness, management and control of hypertension in men and women in Benin, West Africa: the TAHES study

**DOI:** 10.1186/s12872-019-01273-7

**Published:** 2019-12-19

**Authors:** Ileana Desormais, Salimanou Ariyoh Amidou, Yessito Corine Houehanou, Stephan Dismand Houinato, Gwladys Nadia Gbagouidi, Pierre Marie Preux, Victor Aboyans, Philippe Lacroix

**Affiliations:** 1 Department of Thoracic and Vascular Surgery and Vascular Medicine, Dupuytren University Hospital, 2, Ave. Martin Luther King, 87042 Limoges, France; 2 INSERM, Univ. Limoges, CHU Limoges, UMR 1094, Tropical Neuroepidemiology, Institute of Epidemiology and Tropical Neurology, GEIST, Limoges, France; 3 Laboratory of Chronic and Neurological Diseases Epidemiology (LEMACEN), Faculty of Health Sciences, University Abomey-Calavi, Cotonou, Benin; 4 Department of Cardiology, Dupuytren University Hospital, Limoges, France

**Keywords:** Hypertension, General population, Benin

## Abstract

**Background:**

Due to epidemiological transitions, Sub-Saharan Africa is facing a growing burden of non-communicable diseases, including cardiovascular diseases (CVDs). Among their risk factors, hypertension is a major determinant of CVDs, but the prevalence and level of awareness and management of this condition are poorly studied in African populations. The aim of this study was to determine the prevalence of hypertension and identify its associated risk factors as well as the awareness and management of this condition in a community-dwelling cohort in Benin.

**Methods:**

A cross-sectional door-to-door study was conducted in the population over the age of 25 years in Tanve, a rural setting in Benin. The questionnaire and anthropometric measurements of the World Health Organization STEPWISE survey were used. Blood pressure was measured using standard procedures.

**Results:**

The sample included 1777 subjects (60.9% females, mean age was 42.5 ± 16.5 years). The prevalence of hypertension was 32.9%, similar in men (32.8%) and women (33.0%, *p =* 0.9342). Age and obesity were significantly associated with hypertension. Less than half (42%) of hypertensive people were aware about their condition and only 46.3% of them were treated. Awareness ratios differed between men and women (respectively 32.9% vs. 47.5%; *p* = 0.0039) and was not influenced by age, education, occupation, marital status or income. Female sex was the only factor associated with better controlled HTN, independent of socio-economic parameters.

**Conclusion:**

This large population-based study confirms the high prevalence, low awareness, and low control of hypertension in men and women in sub-Saharan Africa. Only half of the populations with hypertension are aware of their hypertension, indicating a high burden of undiagnosed and un-controlled high blood pressure in these populations.

## Background

Hypertension (HTN) is a major risk factor for cardiovascular diseases (CVDs), especially ischemic heart disease and stroke [1]. As the global number of hypertensive people in 2015 was estimated at almost 1.13 billion [2] with a prevalence of HTN within the 30–45% range in adults [3], the global burden of morbidity and mortality related to HTN becomes one of the major public health concerns worldwide. Given its increasing prevalence with advancing age [3] and the trends for population ageing and increased sedentary lifestyle and obesity, the prevalence of HTN is expected to increase even more. It has been predicted that, by 2025, the number of hypertensive individuals will increase by 15–20%, nearing one and a half billion [4]. Although scarce evidence is available in developing countries, the high prevalence of HTN seems consistent across the world, irrespective of the income status (i.e. lower-income, middle-income and higher-income countries) [3]. Few population-based studies indicate that hypertension is also a widespread problem in sub-Sahara Africa (SSA), with a prevalence up to 38% in some communities [5, 6]. It is estimated that out of the approximately 650 million people in SSA, between 10 to 20 million may have hypertension. These estimations are based on scarce heterogenous studies in the past and many countries in SSA still lack detailed recent basic data on the prevalence of HTN [7]. Furthermore, few studies have reported on the proportion of awareness, treatment and good control of HTN in these populations with socio-economic particularities. In Benin, the study conducted in general population in 2008 showed an alarming prevalence of 28% [8]. This condition was highly undiagnosed, as 78% of the subjects were unaware of their high blood pressure.

Our aim was to estimate, using an updated rigorous methodology in accordance to the recent guidelines, the prevalence of hypertension, to identify its associated risk factors and assess the level of awareness and management of hypertension in a rural area of Benin in West Africa.

## Methods

### Study population

This is an analysis of inclusion data collected in the TAnve HEalth Study (TAHES) a prospective population-based cohort study initiated in 2015 in Tanve, a rural setting 150 km north of Cotonou, the capital of Benin. The TAHES involved all adults above 25 years old living in Tanve [9]. Its main objective is to assess the frequency of CVDs and their associated risk factors. Exclusion criteria were pregnant women and refusals to participate.

All participants and/or their families gave informed consent prior to inclusion in the study. Written consent was obtained whenever feasible. For illiterate people, the study’s objectives were verbally explained, and consent was obtained by thumbprint. The ethics committee approved our procedure of thumb print consent.

The study protocol conforms to the ethical guidelines of the 1975 Declaration of Helsinki and had prior approval of the Benin national health’s research ethics committee and the “Comité de Protection des Personnes du Sud-Ouest et d’Outre-Mer 4 in France”.

#### Data collection

An exhaustive sampling using a door-to-door approach was performed. Data were collected by 8 teams of 3 trained investigators, using a questionnaire adapted from WHO STEPS tools [10] in the households. The adapted version has already been used in previous population-based studies in Central Africa [11].

### Sociodemographic data

Sociodemographic data included age, sex, marital status (never married, living with someone as a couple, widow/divorced/separated), education (none, primary education, higher), profession (employee/government employee, craftsman/ storekeeper, farmer/breeder/fisherman, homemade/retired, jobless), and the household income per month (low, middle, high) according to the World Bank indicators [12].

### Cardiovascular risk factors

The cardiovascular risk factors were defined according to the WHO STEPS surveillance manual [13]. Tobacco use was assessed, and participants were classified as never users and current/former users (including cigarette, cigar, pipe or other modes of tobacco use including chewing tobacco).

Weight was measured to the nearest 100 g on mechanical scales (Seca, Hamburg, Germany) and height was measured to the nearest centimetre using a carpenter meter. While the stand upright position was impossible, height was estimated according to the knee height (KH) using Chumlea’s formula for non-Hispanic Black people. Body mass index (BMI) was calculated as weight/height^2^. Underweight was defined as BMI < 18.5 kg/m^2^, normal weight: BMI = 18.5–24.9 kg/m^2^, overweight: BMI = 25–29.9 kg/m^2^, and obese BMI ≥ 30 kg/m^2^.

Diabetes was defined as currently taking antidiabetic drugs or having a fasting capillary whole blood glucose value≥126 mg/dL [14].

### Blood pressure measurements and HTN definition

Systolic (SBP) and diastolic (DBP) blood pressures were recorded, in seated position, after a rest of at least 15 min, using an electronic device (OMRON® M3, OMRON Corporation, Japan). Three measures were recorded, in both arms, at 5-min intervals. In accordance to the 2017 ESC Guidelines on hypertension the average of the last two measurements was used in the analyses and hypertensive subjects was defined by self-reporting ongoing treatment, or SBP ≥140 mmHg and/or DBP ≥90 mmHg [15]. The hypertension was defined as controlled when SBP < 140 mmHg and DBP < 90 mmHg under pharmacological treatment.

### Other data

Nutritional variables included the dietary sodium intake using a food frequency questionnaire [16] and defined according to the WHO guidelines for sodium intake [17].

“Rare” salt intake was defined by low-salt food intake, seasoning less than once a day and consuming ready-made-dishes less than twice a week.

Low intake of fruit and vegetable was defined as consuming less than five total servings (400 g) of fruit and vegetables per day.

Harmful (moderate to heavy) use of alcohol was defined as consumption of > 60 g of alcohol for men or 40 g for women in one occasion within the last 30 day. Consumption below these thresholds was considered as light.

Sedentary lifestyle was defined as < 150 min of moderate-intensity activity (walk, bicycle) per week, or equivalent.

### Data analysis

Descriptive analyses were performed to compare the socio-demographic, cardiovascular risk factors and nutritional variables in men and women. The averages (±SD) and numbers (ratios) were compared with Fisher’s exact test and the Chi-square test, as appropriate.

The association between variables and HTN was assessed by univariable and multivariable analyses. A multivariable logistic regression model was performed to identify associated factors for HTN within demographic variables and CVD risk factors when *p*-value < 0.20 in univariable logistic regression. Interactions between independent variables in the final model were examined.

We performed several models. In these models, age and sex were forced systematically.

First, we adjusted for sociodemographic factors such as age, sex, country, area, marital status, and previous occupation (model 1). Second, cardiovascular risk factors — tobacco use, BMI, physical activity, diabetes — were additionally adjusted (Model 2).

Third, nutritional factors (salt consumption and alcohol consumption, fruits a, d vegetables) were also adjusted for (Model 3).

The level of significance was fixed at 0.05 for all analyses. Statistical analyses were carried out using Statview 5.0 software (SAS Institute, Cary, USA).

## Results

### Study population

Among the 1779 subjects aged 25 years and older who were approached, data for hypertension were missing for 2 participants, resulting in a total sample size of 1777 participants, mean age 42.5 + 16.5 years. Subjects who were 25–34 years old comprised the largest group (41.0%). Females accounted for more than 60% of this population (Table 1).

**Table 1:** Characteristics of study participants

	Total Population n (%)	Men n (%)	Women n (%)	*p* Men vs. Women
Total sample size	1777	695 (39.1)	1082 (60.9)	*0.2732*
Sociodemographic variables				
Age (y)	42.5 ± 16.5	43 ± 16.8	42.1 ± 16.4	*0.2732*
Marital status				*< 0.0001*
Married/in couple	1336 (75.2)	554 (79.7)	782 (72.3)	
Single	213 (12.0)	116 (16.4)	97 (9.0)	
Widowed/divorced/separated	228 (12.8)	25 (3.6)	203 (18.7)	
Education				*< 0.0001*
None	1175 (66.1)	348 (50.1)	827 (76.4)	
Primary	406 (22.9)	216 (31.1)	190 (17.6)	
Higher	196 (11.0)	131 (18.8)	65 (6.0)	
Occupation				*< 0.0001*
Farmer/breeder/fisherman	337 (18.9)	181 (26.0)	156 (14.4)	
Craftsman/storekeeper	1170 (65.8)	385 (55.4)	785 (72.6)	
Employee/government employee	130 (7.3)	93 (13.4)	37 (3.4)	
Homemade/retired	50 (2.8)	6 (0.9)	44 (4.1)	
Jobless	90 (5.1)	30 (4.3)	60 (5.5)	
Religion^a^				*< 0.0001*
Christians	1009 (61.6)	374 (56.4)	635 (65.1)	
Animists	529 (32.3)	254 (38.3)	275 (28.2)	
Other	101 (6.2)	35 (5.3)	66 (6.7)	
Average household income / month				*< 0.0001*
Low	1011 (56.9)	237 (34.1)	774 (71.5)	
Middle	748 (42.1)	447 (64.3)	301 (27.8)	
High	18 (1.0)	11 (1.6)	7 (0.7)	
Depression	171 (9.7)	54 (7.8)	117 (10.8)	*0.0400*
Anxiety	249 (14.0)	63 (9.1)	186 (17.2)	*< 0.0001*
Cardiovascular risk factors				
Body mass index: kg/m^2^	24.2 ± 11.9	23.2 ± 6.5	24.8 ± 5.6	*< 0.0001*
< 18.5	210 (11.9)	97 (14.0)	114 (10.5)	
18.5–25	1032 (58.3)	436 (63.0)	595 (55.1)	
25–30	374 (21.1)	129 (18.6)	245 (22.7)	
> 30	155 (8.7)	30 (4.4)	125 (11.7)	
Tobacco use	152 (8.6)	93 (13.4)	59 (5.5)	*< 0.0001*
Smoked tobacco	100 (5.6)	78 (11.2)	22 (2.0)	*< 0.0001*
Chewed tobacco	76 (4.9)	29 (4.2)	47 (4.3)	*0.0658*
Diabetes^b^	55 (3.1)	21 (3.1)	34 (3.2)	*0.9168*
Physical activity < 150 min/week	1037(58.4)	394 (56.7)	643 (59.4)	*0,2182*
Hypertension	584 (32.9)	228 (32.8)	356 (33.0)	*0.9342*
Nutritional variables				
Fruits and vegetables < 5/day	1603 (90.2)	618 (88.9)	985 (91.0)	*0.1512*
Alcohol consumption				*< 0.0001*
Abstainers	847 (47.7)	196 (28.2)	651 (60.2)	
Light	513 (28.9)	200 (28.8)	310 (28.7)	
Moderate to heavy	417 (23.5)	299 (43.0)	118 (10.9)	
Salt intake (often or very often)^c^	1029 (58.6)	344 (49.7)	685 (63.3)	*< 0.0001*

^a^data were available for 1639 participants

^b^data were available for 1750 participants

^c^data were available for 1755 participants

The global prevalence of hypertension was 32.9% (584 subjects), with the prevalence of hypertension being similar in men and women (32.8% (228) and 33.0% (356) respectively). General characteristics of our study population are detailed in Table 1. The prevalence of hypertension increased significantly with age (OR: 1.04, *p* < 0.001) (Table 2).

**Table 2:** Factors associated with hypertension

Factors	Univariable analysis		Multivariable analysis					
			Model 1^a^		Model 2^b^		Model 3^c^	
	OR (95% CI)	*p*	OR (95% CI)	*p*	OR (95% CI)	*p*	OR (95% CI)	*p*
Age (y)	1.04 (1.03–1.04)	*<0.0001*	1.04 (1.03–1.05)	*<0.0001*	1.04 (1.03–1.05)	*<0.0001*	1.04 (1.03–1.05)	*<0.0001*
Sex								
Males	Ref. 1		Ref. 1		Ref. 1		Ref. 1	
Females	1.00 (0.82–1.23)	*0.9558*	1.07 (0.83–1.38)	*0.5977*	0.94 (0.72–1.22)	*0.6305*	1.01 (0.77–1.33)	*0.9460*
Education								
None	Ref. 1		Ref. 1		Ref. 1		Ref. 1	
Primary	0.59 (0.46–0.76)	*<0.0001*	0.95 (0.71–1.27)	*0.7203*	0.92 (0.68–1.24)	*0.5762*	0.89 (0.66–1.22)	*0.4908*
Higher	0.72 (0.52–1)	*0.0541*	0.70 (0.93–2.28)	*0.1024*	0.81 (0.83–2.12)	*0.2345*	0.80 (0.86–2.19)	*0.1896*
Occupation								
Employee/government employee	Ref. 1		Ref. 1		Ref. 1		Ref. 1	
Craftsman/storekeeper	1.38 (0.90–2.12)	*0.1356*	1.37(0.80–2.34)	*0.2478*	1.29 (0.75–2.24)	*0.3564*	1.27 (0.23-3.12)	*0.3987*
Farmer/breeder/fisherman	2.2 (1.38–3.49)	*0.0008*	1.44 (0.80–2.59)	*0.2216*	1.56 (0.85–2.84)	*0.1501*	1.49(0.81–2.72)	*0.1971*
Homemade/retired	2.84 (1.42–5.66)	*0.0030*	2.31 (1.02–5.22)	*0.0451*	2.79 (1.19–6.53)	*0.0173*	2.81 (1.19–6.67)	*0.0190*
Jobless	5.00 (2.78–8.99)	*<0.0001*	1.52 (0.72–3.20)	*0.2736*	1.73 (0.81–3.70)	*0.1578*	1.61 (0.75–3.46)	*0.2222*
Religion								
Christians	Ref. 1		Ref. 1		Ref. 1			
Animists	1.97 (1.58–2.46)	*<0.0001*	1.43 (1.12–1.82)	*0.0042*	1.57 (1.22–2.02)	*0.0005*	1.52 (1.17–2.62)	*0.0048*
Other	1.22 (0.78–1.89)	*0.3815*	1.06 (0.67–1.69)	*0.8021*	1.12 (0.68–1.77)	*0.6934*	1.16 (0.58–1.89)	0.7834
Marital status								
Single	Ref. 1		Ref. 1					
Married/in couple	1 (0.73–1.38)	*0.9621*	0.89 (0.63–1.26)	*0.5102*				
Widowed/divorced/separated	2.84 (1.92–4.21)	*< 0.0001*	1.10 (0.69–1.77)	*0.6800*				
Average household income/month								
Low	Ref. 1							
Middle	0.97 (0.81–1.21)	*0.8896*						
High	0.78 (0.27–2.20)	*0.6368*						
Depression								
No	Ref. 1							
Yes	1.1 (0.79–1.53)	*0.5790*						
Anxiety								
No	Ref. 1		Ref. 1					
Yes	1.21 (0.91–1.59)	*0.1895*	0.97 (0.71–1.33)	*0.8536*				
Body mass index (kg/m^2^)	1.02 (1.00–1.03)	*0.0002*	1.02 (1.01–1.04)	*0.0001*	1.05 (1.02–1.07)	*0.0001*	1.04 (1.01–1.07)	*0.001*
18.5–25	Ref. 1				Ref. 1		Ref. 1	
< 18.5	1.48 (1.08–2.03)	*0.0141*			1.05 (0.73–1.51)	*0.7940*	1 (0.7–1.46)	*0.9598*
25–30	1.92 (1.50–2.46)	*<0.0001*			2.12 (1.60–2.79)	*<0.0001*	2.10 (1.59–2.18)	*<0.0001*
> 30	2.78 (1.97–3.92)	*<0.0001*			3.57 (2.43–5.25)	*<0.0001*	3.32 (2.25–4.89)	*<0.0001*
Tobacco Use (smoked/chewed)								
No	Ref. 1				Ref. 1			
Yes (past/current)	1.54 (1.10–2.17)	*0.0119*			0.84 (0.57–1.26)	*0.4021*		
Diabetes								
No	Ref. 1							
Yes	1.09 (0.62–1.93)	*0.7533*						
Physical activity								
≥ 150 min/week	Ref. 1				Ref. 1		Ref. 1	
< 150 min/week	1.37 (1.11–1.67)	*0.0027*			1.17 (0.93–1.48)	*0.1755*	1.18 (0.93–1.49)	*0.1725*
Fruits and vegetables								
≥ 5/day	Ref. 1							
< 5/day	1.03 (0.74–1.45)	*0.8363*						
Alcohol consumption								
Abstainers	Ref. 1						Ref. 1	
Light	0.77 (0.60–0.98)	*0.0313*					0.75 (0.57–0.99)	*0.0500*
Moderate to heavy	1.19 (0.94–1.53)	*0.1487*					1.11 (0.82–1.50)	*0.5162*
Salt consumption								
Rarely	Ref. 1						Ref. 1	
Often and very often	1.23 (1–1.50)	*0.0512*					1.11 (0.89–1.41)	*0.3801*

^a^Model 1: adjusted for demographic and socio-economic factors

^b^Model 2: adjusted for demographic, socio-economic and cardiovascular risk factors

^c^Model 3: adjusted for demographic, socio-economic, cardiovascular risk factors and nutritional factors

The univariable analysis indicated that increased age, the past or current use of tobacco, increased BMI, sedentary lifestyle, widowed or divorced status, previous occupation other than employee, lower education, animist religion, and increased salt consumption were associated with an increased likelihood of hypertension (Table 2). In the full model (model 3), increased age and BMI, homemade and retired status as well as animist religion remained significantly associated with an increased likelihood of hypertension, whereas the moderate alcohol consumption remained associated with a lower ratio of HTN (Table 2). No interactions between independent variables have been found.

Further analyses suggested a higher likelihood of hypertension in the case of high to very high salt consumption and sedentary lifestyle in women as compared to men who seemed less exposed to HTN by a moderate alcohol intake (Table 3). Age, increasing BMI and animist religion remain significant associated HTN factors in both men and women.

**Table 3:** Factors associated with hypertension in male and female population

Factors	FEMALES		MALES	
	Multivariable analysis^a^			
	OR (95% CI)	*p*	OR (95% CI)	*p*
Age (y)	1.05 (1.04–1.06)	*<0.0001*	1.03 (1.01–1.04)	*<0.0001*
Marital status				
Single	Ref. 1		Ref. 1	
Married/in couple	0.92 (0.54–1.55)	*0.7499*	0.86 (0.52–1.45)	*0.5752*
Widowed/divorced/separated	0.98 (0.52–1.85)	*0.9547*	1.38 (0.50–3.81)	*0.5280*
Education				
None	Ref. 1		Ref. 1	
Primary	0.87 (0.56–1.37)	*0.5583*	0.91 (0.59–1.41)	*0.6763*
Higher	1.32 (0.58–3.00)	*0.5038*	1.21 (0.65–2.34)	*0.5519*
Occupation				
Employee/government employee	Ref. 1		Ref. 1	
Craftsman/storekeeper	0.98 (0.33–2.93)	*0.9745*	1.21 (0.63–2.34)	*0.5706*
Farmer/breeder/fisherman	1.30 (0.41–4.16)	*0.6550*	1.37 (0.64–2.90)	*0.4169*
Homemade/retired	2.06 (0.55–7.73)	*0.2895*	7.19 (0.75–69.29)	*0.0876*
Jobless	1.46 (0.40–5.34)	*0.5638*	0.85 (0.26–0.74)	*0.7804*
Religion				
Christians	Ref. 1		Ref. 1	
Animists	1.52 (1.06–2.16)	*0.0211*	1.75 (1.17–2.62)	*0.0068*
Other	0.94 (0.51–1.73)	*0.8408*	1.72 (0.76–3.89)	*0.1921*
Depression				
No	Ref. 1		Ref. 1	
Yes	1.94 (0.54–1.66)	*0.8444*	1.55 (2.56–1.19)	*0.1277*
Anxiety				
No	Ref. 1		Ref. 1	
Yes	0.87 (0.57–1.40)	*0.5789*	1.77 (0.91–3.42)	*0.0922*
Body mass index (kg/m^2^)				
18.5–25	Ref. 1			
< 18.5	0.56 (0.31–0.99)	*0.0482*	1.29 (0.75–2.22)	*0.3593*
25–30	2.16 (1.49–3.12)	*< 0.0001*	1.89 (1.19–3.02)	*0.0069*
> 30	3.02 (1.89–4.81)	*< 0.0001*	5.28 (2.36–11.77)	*< 0.0001*
Tobacco				
No	Ref. 1			
Yes	0.75 (0.39–1.46)	*0.4032*	0.83 (0.57–1.19)	*0.4916*
Diabetes				
No	Ref. 1		Ref. 1	
Yes	0.83 (0.34–2.00)	*0.6723*	1.43 (0.53–3.86)	*0.4761*
Physical activity				
≥ 150 min/week	Ref. 1		Ref. 1	
< 150 min/week	1.44 (1.05–1.95)	*0.0245*	0.99 (0.68–1.44)	*0.9520*
≥ 5/day	Ref. 1		Ref. 1	
< 5/day	0.95 (0.55–1.65)	*0.8567*	1.13 (0.61–2.10)	*0.6993*
Alcohol consumption				
Abstainers	Ref. 1		Ref. 1	
Light	0.73 (0.51–1.04)	*0.0838*	0.74 (0.45–0.98)	*0.0467*
Moderate to heavy	1.05 (0.64–1.73)	*0.6723*	1.14 (0.73–1.78)	*0.5732*
Salt consumption				
Rarely	Ref. 1		Ref. 1	
Often or very often	1.42 (1.02–1.97)	*0.0365*	0.83 (0.57–1.20)	*0.3168*

^a^Adjusted for demographic and socio-economic, cardiovascular risk factors and nutritional factors

*Awareness, Treatment, and Control ratios of hypertension.*

**Fig. 1:**
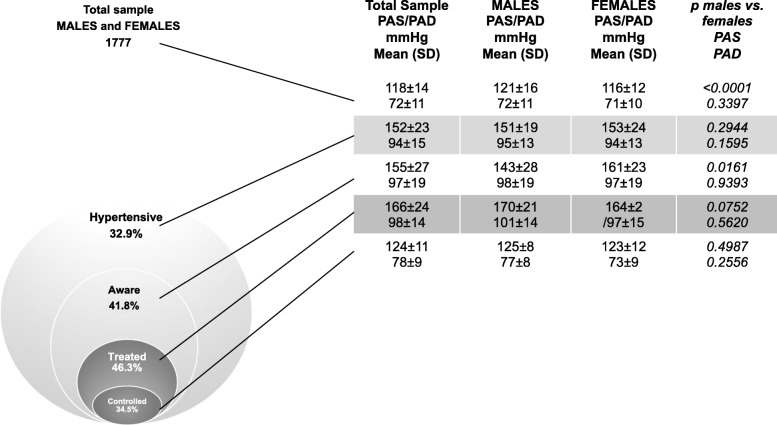
Venn diagram representing the prevalence and the proportions of awareness, treatment and controlled hypertension in study population. For each category, values of systolic (SBP) and diastolic (DBP) blood pressures in both sexes

**Fig. 2:**
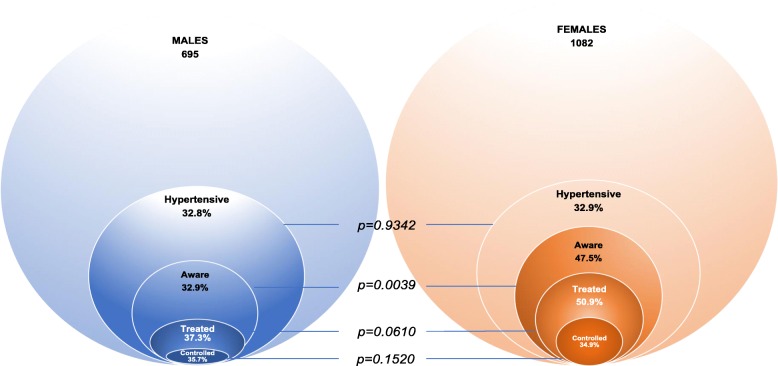
Venn diagrams representing the prevalence and the proportions of awareness, treatment and controlled hypertension in males and females

Among hypertensive participants, less than one-half (41.8%) were aware of their condition (Fig. 1). Awareness ratios differed between men and women (32.9% vs. 47.5%; *p* = 0.0039) (Fig. 2) and was not influenced by age, education, profession, marital status or income. Female sex was the only associated factor with controlled HTN, independently of the education, occupation, income or marital status (Table 4).

**Table 4:** Factors associated with controlled hypertension

Factors	Multivariable analysis	
	OR (95% CI)	*p*
Age (y)	0.98 (0.96–1.01)	*0.2992*
Sex		
Males	Ref. 1	
Females	2.90 (1.11–5.57)	*0.0296*
Education		
None	Ref. 1	
Primary	0.57 (0.18–1.79)	*0.3358*
Higher	1.39 (0.37–5.2)	*0.6273*
Occupation		
Employee/government employee	Ref. 1	
Craftsman/storekeeper	0.67 (0.14–3.18)	*0.6177*
Farmer/breeder/fisherman	0.54 (0.09–3.36)	*0.5103*
Homemade/retired	0.54 (0.05–6.69)	*0.6279*
Jobless	0.40 (0.03–5.90)	*0.5040*
Religion		
Christians	Ref. 1	
Animists	0.90 (0.38–2.10)	*0.7997*
Other	1.81 (0.47–7.01)	*0.3886*
Marital status		
Single	Ref. 1	
Married/in couple	1.14 (0.36–3.60)	*0.8216*
Widowed/divorced/separated	0.43 (0.08–2.31)	*0.3197*
Average household income/month		
Low	Ref. 1	
Middle/high	1.24 (0.56–2.73)	*0.5899*

Only 46.3% (113) of hypertensive participants declared receiving antihypertensive treatment at the time of the study (Fig. 1). No difference in the prevalence of treatment was observed between men and women (37.3% vs. 50.3%, *p = 0.0610*), nor in uncontrolled hypertensive (34.1% vs. 35.7%, *p = 0.1520*). Treatment was not influenced by the socio-economic status.

## Discussion

To our knowledge, this population-based study, conducted following the STEPS method recommended by the WHO for screening and monitoring risk factors of noncommunicable diseases, is the one of the largest investigations in the past 5 years on the prevalence of HTN and associated factors in rural population in West Africa. The study on hypertension, conducted in 2008, in Benin, showed an alarming prevalence in general population (27.9%) [8]. This condition was highly undiagnosed, as 78% of the subjects were unaware of their high blood pressure. As hypothesized, our study underlines the highly increased HTN prevalence (32.9%) since 2008 [8]. Previous studies also suggested a higher prevalence of HNT in urban areas compared to rural zones, explained by the fact that rural African populations were characterized by a traditional lifestyle associating cultural and dietary habits more in favour of HTN prevention (27.1% in rural areas vs 29.5% in urban zones in 2011) [18]. Almost 20 years later, our study points out the sociocultural transition in rural areas with a prevalence of HNTN even higher than the prevalence described previously in urban population. The prevalence of HTN observed in the present study is comparable to the recently reported prevalence in Ghana, Nigeria, South Africa, Sudan and Tanzania [19–21]. The progressive aging of the population with the increase in life expectancy may partially explain the progression of HTN prevalence in African countries.

The factors generally associated with hypertension include age, BMI, sedentary lifestyle, tobacco use, alcohol consumption. Most of these have previously been identified as important risk factors for hypertension in different studies conducted in SSA [11, 22–26]. Our results also confirm some of these observed associations such as increasing age, sedentary lifestyle and BMI. Our study observed that tobacco users, generally considered at higher risk of CVD were not at higher risk to be hypertensive. This result is probably due to the very low ratio of tobacco use in Benin. Also, alcohol consumption, a well-known risk factor of HTN [15] was not significantly reported in our analyses. In other sub-Saharan African studies results are also controversial, showing either association, or no significant association between HTN and moderate to heavy alcohol consumption [27, 28]. Furthermore, our study suggests a slightly protective effect of the light alcohol consumption in men, probably as the reflect of a healthy lifestyle. These discrepancies in hardly comparable results might be explained by the use of different settings, and assessments of alcohol consumption from one study to another.

Recent data show that salt consumption around the world is much higher than is physiologically necessary [29], even more in males than females at any age [30, 31]. In a systematic review and meta-regression in 2016 no gender difference was reported in African studies [32]. In our study, high salt intake, was independently associated with a higher likelihood of prevalent hypertension. Dietary estimates of sodium intake might not be accurate due to recall bias, reporting errors, erroneous food composition tables (especially if not country-specific or outdated), and/or difficulty in quantifying added salt (e.g. salt added during cooking but discarded in cooking water, etc). However, the fact that there might be a difference between men and women, suggests that context-specific research is needed to establish whether patterns of sodium intake in the SSA settings differ from high-income countries or other low- or middle-income countries.

Our finding regarding higher ratios of HTN in subjects of animist religion are intriguing and we do not find any rational as declarative questionnaires on lifestyle used in our study were not exhaustive. Previous studies suggested that religiosity could be a factor involved in the management of health and diseases/patient longevity [33]. Nevertheless, further specific confirmatory and exploratory studies are needed.

In our study, less than 42% of hypertensive participants were aware of their pathology and less than one-half of them took antihypertensive treatments. Among treated subjects, only one-third had controlled hypertension. Few studies have analysed awareness and treatment ratios of hypertension, as well as the fraction with controlled hypertension in Africa [8, 11].

The ratios of awareness and controlled hypertension were higher than other studies on the continent. These differences might be related to variances in socioeconomic levels, healthcare access and preventive measures to reduce HTN. The findings of our study also suggest that independently of the socio-cultural status, women could be more receptive to treatment and more regular during the follow up. This could be explained by a better medical survey for women during and after pregnancy.

Our study has several limitations. Although the definition of hypertension respects the current guidelines, no ambulatory pressures were available. Furthermore, the cross-sectional design of the study, do not allow the assessment of the temporal nature of the associations and survival bias cannot be totally excluded. We also cannot exclude recall bias, especially for nutritional factors, but this it is limited since informants were interviewed alongside participants. Also, the 24-h urinary salt excretion was not available. Assessment of dietary sodium intake used a food frequency questionnaire which might underestimate the sodium intake by 30 to 50%. Data on dyslipidaemia were lacking and thus were not adjusted for in this study.

Despite these limitations, our study remains the largest study in general African population using a rigorous method of HTN assessment according to the latest guidelines and brings important epidemiological data on HNT in developing countries.

More studies are needed in the future to identify reasons behind increased prevalence and poor blood pressure control and examine trends in prevalence, awareness, treatment, and control. TAHES cohort will go on during the years to come in order to analyse the impact of an educational program, based on local culture and economic characteristics, on the management and control of HTN in this population.

The role of new tools for hypertension management will also be tested.

The aim of the TAHES cohort is to provide epidemiological data and propose ways of improvement in HTN management in order to promote preventive programmes aiming to increase public awareness and educate physicians.

## Conclusions

In terms of public health, this large population-based study confirms the high burden of HTN in sub-Saharan Africa and highlights the urgent need for HTN screening, treatment, and control. Less than half of the population with hypertension are aware of their hypertension, indicating the burden of undiagnosed and un-controlled high blood pressure in these populations [34]. Future studies in the same setting would enable to assess the evolution of hypertension and its management in this specific setting.

## Data Availability

The datasets used and/or analysed during the current study are available from the corresponding author on reasonable request.

## Abbreviations

BMI: Body mass index
CVDs: Cardiovascular diseases
DBP: Diastolic blood pressure
HTN: Hypertension
KH: Knee height
SBP: Systolic blood pressure
SSA: Sub-Sahara Africa
TAHES: TAnve HEalth study
